# Hybrid Artificial Neural Network-Based Models to Investigate Deformation Behavior of AZ31B Magnesium Alloy at Warm Tensile Deformation

**DOI:** 10.3390/ma16155308

**Published:** 2023-07-28

**Authors:** Mohanraj Murugesan, Jae-Hyeong Yu, Wanjin Chung, Chang-Whan Lee

**Affiliations:** 1 Department of Mechanical System Design Engineering, Seoul National University of Science and Technology, Seoul 01811, Republic of Korea; mohanaero45@seoultech.ac.kr (M.M.); wjchung@seoultech.ac.kr (W.C.); 2 Department of Mechanical Information Engineering, Seoul National University of Science and Technology, Seoul 01811, Republic of Korea; jhyu9109@seoultech.ac.kr

**Keywords:** AZ31 magnesium alloy, warm tensile experiments, flow stress, artificial neural network, genetic algorithm, constrained nonlinear function, finite element analysis

## Abstract

The uniaxial warm tensile experiments were carried out in deformation temperatures (50–250 °C) and strain rates (0.005 to 0.0167 s${}^{-1}$) to investigate the material workability and to predict flow stress of AZ31B magnesium alloy. The back–propagation artificial neural network (BP–ANN) model, a hybrid models with a genetic algorithm (GABP–ANN), and a constrained nonlinear function (CFBP–ANN) were investigated. In order to train the exploited machine learning models, the process parameters such as strain, strain rate, and temperature were accounted as inputs and flow stress was considered as output; moreover, the experimental flow stress values were also normalized to constructively run the neural networks and to achieve better generalization and stabilization in the trained network. Additionally, the proposed model’s closeness and validness were quantified by coefficient of determination ($R^{2}$), relative mean square error (RMSE), and average absolute relative error (AARE) metrics. The computed statistical outcomes disclose that the flow stress predicted by both GABP–ANN and CFBP–ANN models exhibited better closeness with the experimental data. Moreover, compared with the GABP–ANN model outcomes, the CFBP–ANN model has a relatively higher predictability. Thus, the outcomes confirm that the proposed CFBP–ANN model can result in the accurate description of AZ31 magnesium alloy deformation behavior, showing potential for the purpose of practicing finite element analysis.

## 1. Introduction

Magnesium alloys find extensive use in numerous industries like automobile, aerospace, electronics, and transportation considering their advantageous properties. These alloys possess a combination of low density, high specific strength, dimensional stability, high damping capacity, thermal conductivity, electromagnetic shielding capabilities, and the potential for recyclability [1,2]. However, at room temperature, the formability of magnesium alloys is limited by its hexagonal close-packed structure, which primarily allows for basal slip activation [2,3]. These features present challenges for further advancements and broader applications of magnesium alloys. To overcome these limitations and improve the material formability, it is important to investigate the flow stress behavior of magnesium alloys during hot deformation. Such investigations aid in understanding the material’s deformation characteristics and pave the way for enhancing its formability [3,4,5]. The constitutive models are extensively used in material processing to describe material behavior, considering work hardening, strain rate sensitivity, and thermal softening at elevated temperatures and strain rates [6,7]. Understanding the ductile materials behavior is crucial for accurate material description with finite element (FE) tools as it forms the basis of numerical simulations to simulate the material’s thermal-mechanical behavior effectively [8,9]. Employing constitutive models is motivated by systematically determining model parameters through fitting measured flow stress values. This approach enables the accurate characterization of material flow behavior [10,11] because a reliable flow stress model supports the prediction of material ductility across various deformation temperatures and strain rates in design field. Despite various categories like physically-based, semi–empirical, and empirical, the flow stress model’s intention remains the same, which is to accurately forecast the specific material behavior under various processing conditions [11,12].

Many researchers have aimed to develop a precise constitutive model to forecast the material deformation behavior using the phenomenological-based constitutive models [13,14,15]. Johnson and Cook suggested a Johnson–Cook (JC) constitutive model accounting for strain effect, strain hardening effect, and temperature effect independent of each other for capturing different material’s deformation behavior at higher strains, elevated strain rates, and deformation temperatures. On the other hand, as the JC model only accounts for the independent response of strain, strain rate sensitivity, and temperature, the JC model might lose the predictability at accounted deformation temperatures and strain rates [16,17,18]. For example, Liang et al. [14] suggested a constitutive model using the original JC model and the modified JC model to foresee the Al–Si–Mg alloy flow behavior and discussed that the modified JC model prediction was close to the test data in accurately describing the Al–Si–Mg alloy flow behavior. The authors, Song et al. [15], Li et al. [16], Wang et al. [17], Tan et al. [18], and Murugesan et al. [19] also proposed the modified JC models, mainly for titanium, steel, nickel, and aluminum alloys, considering the combined responses of strain rate and temperature upon the flow stress; their results from the flow stress prediction agreed well with the experimental observations for the tested materials.

Furthermore, Zerilli and Armstrong proposed a Zerilli–Armstrong (ZA) constitutive model based on the dislocation mechanism for materials such as copper and iron and discussed that the constitutive model yielded different model parameters for dissimilar materials because of inhomogeneous dislocation properties [20]. Even though it is based on dislocation mechanics, the modified ZA model parameters can be calculated as same as the original JC and the modified JC models; thus, the modified ZA model is yet called a semi-empirical model. For example, Zhan et al. [21] proposed constitutive models for the Ti–6Cr–5Mo–5V–4Al alloy based on modified JC and ZA models and discussed that the modified ZA model showed more significant accuracy in the predictability than the modified JC model. Similarly, the authors, He et al. [22], Han et al. [23], Li et al. [24], and Murugesan et al. [25], discussed that exploiting the Arrhenius–type constitutive equation, which considers the strain–compensation into the flow stress, could result in accurate forecast of flow stress against the test data. It is obvious that the material response is highly nonlinear under higher strain rates and temperatures, and the factors influencing the flow stress are also nonlinear in most cases. This could result in the inaccurate prediction of flow stress because of the improper estimation of statistical model parameters due to the non–linearity and outliers present in the flow stress data.

Recently, a countable number of researchers effectively used a new approach, called the back–propagation artificial neural network (BP–ANN) model, to propose the constitutive relationships in a prediction of flow stress, notably for complex and nonlinear problems [22]. Unlike the phenomenological models, the BP–ANN model identifies the material behavior by learning from the model training and understands the presence relationship among the inputs and outputs outside of any prior hypotheses about its nature and interrelationships [24]. Xiao et al. [26] conducted a comparative study about the Arrhenius–type constitutive equation and BP–ANN model in a prediction of 12Cr3WV steel deformation behavior and discussed that 12Cr3WV steel flow behavior can be more accurately captured by the optimized BP–ANN model than the Arrhenius-type constitutive model. Likewise, the constitutive relationships were proposed for various materials using the BP–ANN models by researchers Li et al. [27], Stendal et al. [28], WD et al. [29], Thakur et al. [30], and Murugesan et al. [31] and stated that the neural network model could be an impressive tool to examine the deformation behavior and to suggest the constitutive equation of test materials. However, the BP–ANN model can yield different results at each run due to random outcomes of weights and bias in the neural network and is often termed a multi–restart problem [31]. Even though a reasonable number of studies were reported related to the constitutive modeling of the AZ31B magnesium alloy, the prediction accuracy was not completely achieved from the phenomenological models [32] , which shows the importance of exploiting the BP–ANN models to propose the constitutive model in a prediction of AZ31B magnesium alloy deformation behavior [1,2,3,4,5]. Moreover, choosing or designing an optimum neural network with a reasonable number of hidden layers and optimum weights and biases is quite challenging in complex nonlinear problems [31,33]. In this work, the procedure to estimate the minimum number of hidden layers and the minimum number of samples to train the network is presented. Additionally, a hybrid BP–ANN model with a genetic algorithm and a constrained nonlinear function is proposed for describing the material deformation behavior.

This work aims to establish a highly accurate flow stress model to forecast the material flow behavior of AZ31 magnesium alloy material. The uniaxial warm tensile experiments were carried out in three rolling directions (RDs): 0°, 45°, and 90°, in deformation temperatures of 50–250 °C and strain rates of 0.005–0.0167 s${}^{-1}$ to investigate the material workability and to compute the flow stress of AZ31B magnesium alloy material. The back–propagation artificial neural network (BP–ANN) model, a hybrid model with a genetic algorithm (GABP–ANN), and a constrained nonlinear function (CFBP–ANN) were investigated. Additionally, the proposed model’s closeness and validness were quantified by the coefficient of determination ($R^{2}$), relative mean square error (RMSE), and average absolute relative error (AARE) metrics.

## 2. Materials and Experimental Procedures

**Figure 1:**
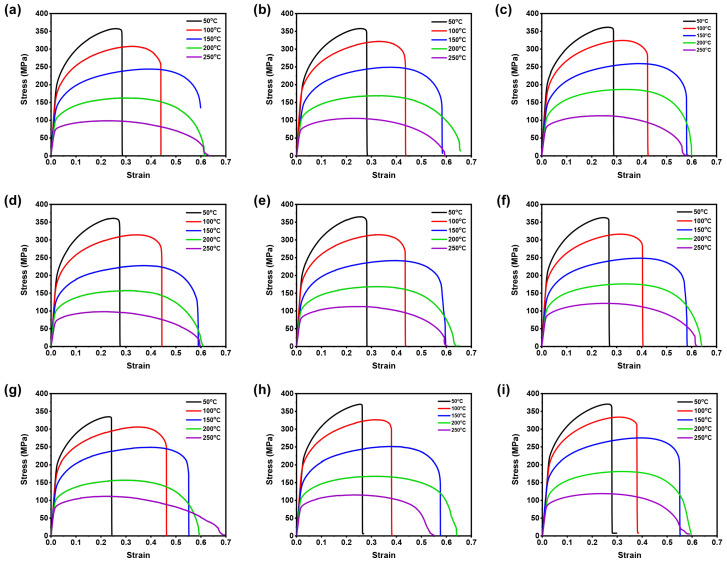
True strain–stress data received from (**a**) 0.005 s${}^{-1}$, (**b**) 0.01 s${}^{-1}$, and (**c**) 0.0167 s${}^{-1}$ at 0° RD; (**d**) 0.005 s${}^{-1}$, (**e**) 0.01 s${}^{-1}$, and (**f**) 0.0167 s${}^{-1}$ at 45° RD; and (**g**) 0.005 s${}^{-1}$, (**h**) 0.01 s${}^{-1}$, and (**i**) 0.0167 s${}^{-1}$ at 90° RD.

The test material in this work is AZ31B magnesium alloy material, and its chemical composition (in wt.%) is listed in Table 1 [32]. The flat test samples of 6 mm in width and 12 mm gauge length were made from 1 mm–thick AZ31B magnesium alloy sheets at three rolling directions (RDs): 0°, 45°, and 90°, according to the ASTM–E8M subsize standard for conducting the warm tensile tests at temperatures (50–250 °C) and strain rates (0.005–0.0167 s${}^{-1}$). After performing each experiment, the load–displacement test data were obtained, and then the stress–strain (SS) data were averaged and converted into true SS using general equations [32]. Figure 1 illustrates the true flow SS curves received from the warm tensile experiments at accounted test conditions with respect to three rolling directions. Figure 1 confirms that temperatures and strain rates have a meaningful impact on flow stress. For example, the flow stress increases significantly at lower temperature, and on the contrary, the flow stress also decreases with the increase in the temperature [32]. Moreover, from flow curves, it is evident that the significant work hardening occurred at tested strain rates up to 200 °C, whereas the flow stress curves from 250 °C for tested strain rates show the dynamic softening behavior at the end after a small work–hardening stage at the beginning [32]. Further, the plastic true SS data will be utilized to construct the neural network models.

## 3. Proposed Flow Stress Models for Describing Hot Deformation Behavior

### 3.1. Development of Artificial Neural Network Model with Backpropagation (BP–ANN) Algorithm

**Figure 2:**
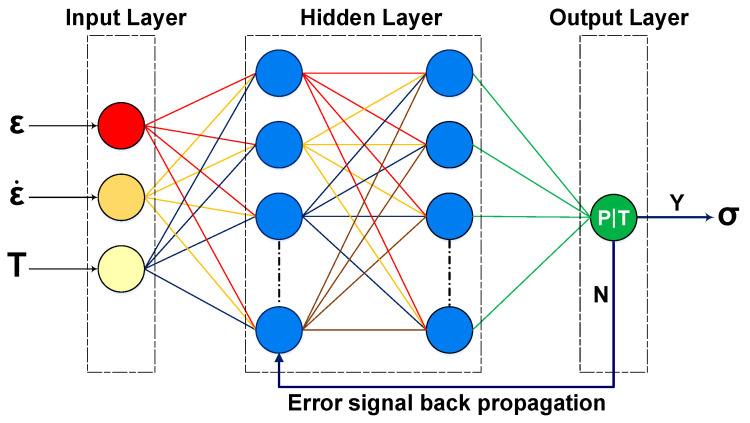
Schematic representation of back–propagation artificial neural network model [31].

The feed forward back–propagation artificial neural networks (BP–ANNs) are commonly structured with an input layer, an output layer, and a hidden layer, as illustrated in Figure 2. They learn from examples and are capable of recognizing patterns in input and output data sets outside of any prior assumptions about the relationships between them. The back–propagation algorithm is commonly used in an ANNs model to adjust the weights and biases [31,33,35]. It operates by approximating these adjustments in a vector space and employs gradient descent to minimize the error. Thus, this ability allows ANNs to minimize the error (Equation (1)) between the predicted output and the known test targets at the time of the training process. Because of these advantages, ANNs have gained popularity in predicting and modeling the metallic materials hot deformation behavior at elevated deformation temperature and strain rates. Therefore, as shown in Figure 2, a three–layer BP–ANN model was used in this work to evaluate AZ31B magnesium alloy deformation behavior. The BP–ANN model inputs were assumed as strain (ε), strain rate ($\dot{\varepsilon }$), and deformation temperature (*T*), and flow stress (σ) was considered as a target.

(1) $$\begin{array}{c} \begin{array}{c} \mathrm{MSE}=\frac{1}{n}\sum_{i=1}^{n}{\left(E_{i}-P_{i}\right)}^{2}\;n=\mathrm{total}\;\mathrm{no}.\;\mathrm{of}\;\mathrm{samples} \end{array} \end{array}$$

Carpenter et al. [36] recommended that the architecture of the BP–ANN model can be devised using the following information:

$$\begin{array}{c} \begin{array}{c} \begin{array}{cc} \mathrm{Hidden}\;\mathrm{layer}\;\mathrm{neurons}\;\left(\mathrm{HN}\right) & =\mathrm{Input}\;\mathrm{layer}\;\mathrm{variables}\;\left(\mathrm{IN}\right)+1\\ \mathrm{HN} & =3+1=4 \end{array} \end{array} \end{array}$$

Moreover, in order to train the feed forward BP–ANN model, a minimum number of required samples can be determined as below [31,36,37]:

$$\begin{array}{c} \begin{array}{c} \begin{array}{cc} \mathrm{No}.\;\mathrm{of}\;\mathrm{training}\;\mathrm{data}\;\left(\mathrm{NT}\right) & =\mathrm{HN}*(\mathrm{input}\;\mathrm{layer}\;\mathrm{variables}\;\left(\mathrm{IN}\right)+1)+\\ \; & \mathrm{Output}\;\mathrm{layer}\;\mathrm{variables}\;\left(\mathrm{NO}\right)*(\mathrm{HN}+1)=21 \end{array} \end{array} \end{array}$$

In this present BP–ANN model, 345 datasets were selected from the plastic true SS curves (Figure 1), and the datasets were divided into three categories such as training dataset (60%), validation dataset (15%), and test dataset (25%). The datasets were extracted from the strain range of 0.03 to 0.25 at the interval of 0.01 and used to train the proposed BP–ANN model. In the BP–ANN model, the transfer functions were selected as tan sigmoid (hidden layer) and purelin (output layer), and the trainlm (Levenberg–Marquardt algorithm) was chosen as training function to train the model [31]. For example, at first, the dataset received from 0° RD was used to train the neural network. Then, in order to effectively run the neural network, both input and output parameters were normalized between 0 to 0.95 using Equation (2). To determine the appropriate number of neurons for the hidden layer, an initial trial and error approach was employed, starting with four neurons. Subsequently, the number of neurons was gradually increased in the neural network [31,33]. From the results, it was observed that the neural network with one hidden layer aggregating four neurons resulted in good predictability with considerable prediction error. Therefor, the same neural network structure was used to solve the remaining test cases, such as 45° and 90° RDs, to evaluate the deformation behavior of AZ31B magnesium alloy.

(2) $$\begin{array}{c} \begin{array}{c} X_{N}=\frac{X-0.95X_{\min}}{1.05X_{\max}-0.95X_{\min}} \end{array} \end{array}$$

where *X*, $X_{\min}$, $X_{\max}$, and $X_{N}$ are the test data, minimum and maximum of test data, and normalized test data, respectively [31].

### 3.2. Development of BP–ANN Model with a Genetic Algorithm

**Figure 3:**
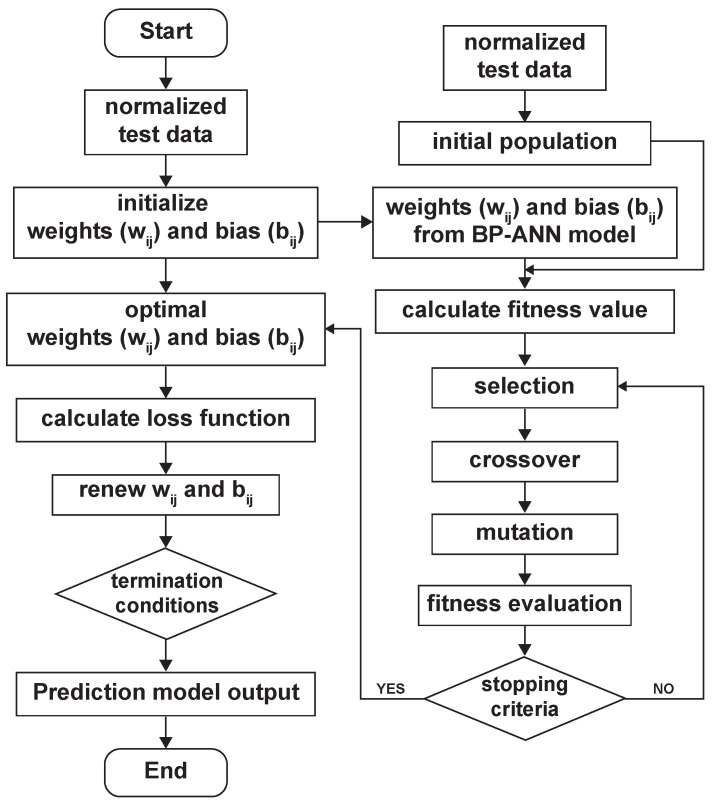
Flow chart of the BP–ANN model combined with a genetic algorithm.

Genetic algorithms (GAs) are iterative optimization algorithms inspired by natural selection and genetics. They find approximate solutions to complex problems. GAs evolve a population of individuals represented as chromosomes. Each individual is a possible solution encoded as symbol strings [38]. Moreover, the GA operates through several steps: generating an initial population, evaluating individuals using a fitness function, selecting the fittest individuals for reproduction, combining individuals through crossover and mutation to create offspring, and repeating these steps over generations to improve the population’s overall fitness. In addition, GAs excel at global searching and at exploring diverse solutions simultaneously to avoid local optima. They can be parallelized, evaluating and evolving multiple individuals concurrently, which speeds up optimization, particularly for computationally intensive problems [38]. It is obvious that ANNs have drawbacks such as local minima and network paralysis, which can hinder their ability to adjust weights effectively and can lead to reduced system accuracy. Similarly, relying solely on a genetic algorithm may not always yield optimal solutions. To address these challenges, a hybrid approach (GABP–ANN) combining the BP–ANN model and GA is proposed in this work to evaluate AZ31B magnesium alloy deformation behavior as shown in Figure 3 because GAs can be used to optimize the parameters or architecture of neural networks, including the weights and biases of individual neurons. By combining the global search capabilities of GAs with the learning ability of neural networks, it is possible to enhance the performance and overcome the limitations of traditional optimization methods for neural networks [38].

Similar to the BP–ANN model, as discussed in Section 3.1, the same datasets, such as training (60%), validation (15%), and test (25%), were exploited for training the proposed hybrid GABP–ANN model. Further, the BP–ANN model was optimized using the genetic algorithm considering bounds constraints, and in order to receive a more systematic solution, the solver options such as max generations, function tolerance, and constraint tolerance were chosen as 50, 1 $\times \;10^{-10}$, and 1 $\times \;10^{-10}$, respectively.

### 3.3. Development of BP–ANN Model with a Constrained Nonlinear Function

**Figure 4:**
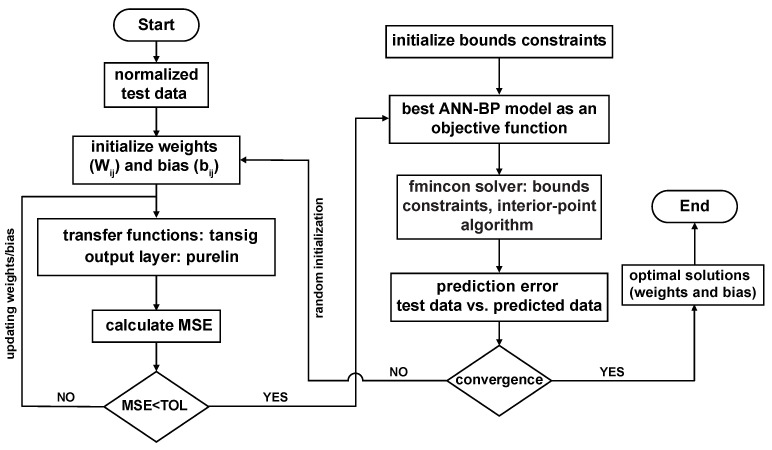
Flow chart of the BP–ANN model combined with a constrained nonlinear function [31].

In this study, a hybrid optimization method with a constrained nonlinear function (CFBP–ANN) was also proposed to train a network model for achieving the optimum weights and biases to predict the flow stress of AZ31B magnesium alloy, as shown in Figure 4. To minimize the prediction error, the fmincon function, which is based on the interior–point (IP) algorithm, was employed. Like for the GABP–ANN model, here the bounds–constrained optimization techniques were also implemented to handle function minimization within the constraints of the CFBP–ANN model. By using fmincon instead of GAs, the computational time can be reduced while maintaining accuracy. The determination of the specific bounds constraints was influenced by previous trial experiences. The common format of optimization steps can be outlined as follows [31]:

$$\begin{array}{cc} \underset{x}{\mathrm{Minimize}\text{:}\;}\\ \; & \;\;\mathrm{AARE}=\frac{1}{n}\sum_{i=1}^{n}\left|\frac{\sigma_{e}^{i}-\sigma_{A}^{i}}{\sigma_{e}^{i}}\right|\times 100\%,\\ \; & \mathrm{where}\;\sigma_{A}\;\mathrm{from}\;\mathrm{the}\;\mathrm{best}\;\mathrm{BP}–\mathrm{ANN}\;\mathrm{model}\\ \mathrm{subjected}\;\mathrm{to} & \left\{\begin{array}{c} \;{\mathrm{IW}}_{\mathrm{lb}}\leq x\left(1\right)\leq {\mathrm{IW}}_{\mathrm{ub}}\\ \;{\mathrm{LW}}_{\mathrm{lb}}\leq x\left(2\right)\leq {\mathrm{LW}}_{\mathrm{ub}}\\ \;{b1}_{\mathrm{lb}}\leq x\left(3\right)\leq {b1}_{\mathrm{ub}}\\ \;{b2}_{\mathrm{lb}}\leq x\left(4\right)\leq {b2}_{\mathrm{ub}} \end{array}\right. \end{array}$$

### 3.4. Model Validation and Verification

The proposed constitutive equations predictability is further verified through employing standard statistical parameters such as $R^{2}$, and RMSE as follows [25,31,39,40]:

(3) $$R^{2}=1-\frac{\sum_{i=1}^{n}{\left(\sigma_{e}^{i}-\sigma_{p}^{i}\right)}^{2}}{\sum_{i=1}^{n}{\left(\sigma_{e}^{i}-{\bar{\sigma }}_{e}\right)}^{2}},$$

(4) $$\mathrm{RMSE}=\sqrt{\frac{1}{n}\sum_{i=1}^{n}{\left(\sigma_{e}^{i}-\sigma_{p}^{i}\right)}^{2}}$$

The coefficient $R^{2}$ indicates the level of linear association between the experimental and predicted data, while the RMSE data provide a means to compare relative errors on a term–by–term basis. Likewise, the prediction error between test data and calculated data can be determined using the following calculation [25,31,39,40]:

(5) $$\mathrm{AARE}=\frac{1}{n}\sum_{i=1}^{n}\left|\frac{\sigma_{e}^{i}-\sigma_{p}^{i}}{\sigma_{e}^{i}}\right|\times 100\%,$$

where $\sigma_{e}$, $\sigma_{p}$, and *n* are the experimental true stress, the predicted true stress, and the total number of true stress data, respectively.

## 4. Results and Discussion

**Figure 5:**
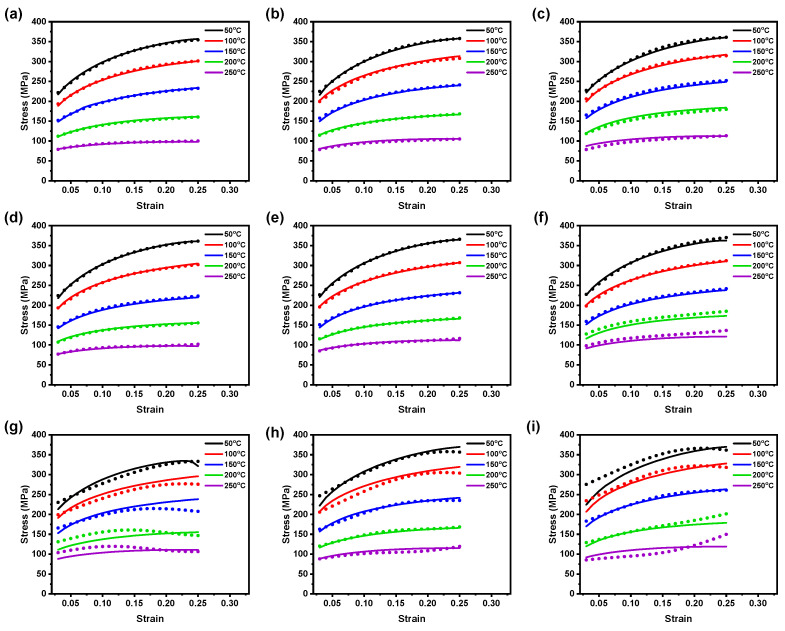
Comparison plot between test data (lines) vs. calculated data (dots) by the BP–ANN model at $\dot{\varepsilon }$ of (**a**) 0.005 s${}^{-1}$, (**b**) 0.01 s${}^{-1}$, and (**c**) 0.0167 s${}^{-1}$ from 0° RD; (**d**) 0.005 s${}^{-1}$, (**e**) 0.01 s${}^{-1}$, and (**f**) 0.0167 s${}^{-1}$ from 45° RD; and (**g**) 0.005 s${}^{-1}$, (**h**) 0.01 s${}^{-1}$, and (**i**) 0.0167 s${}^{-1}$ from 90° RD.

**Figure 6:**
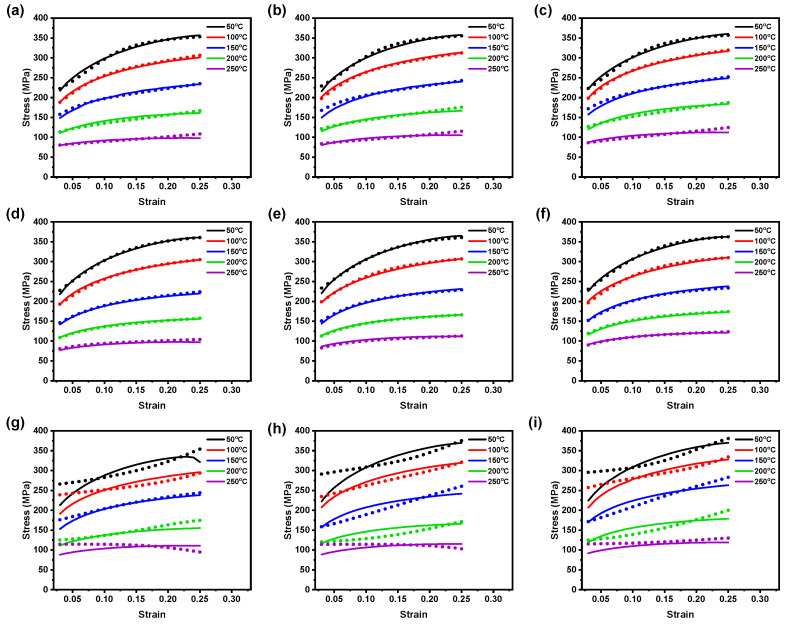
Comparison plot between test data (lines) vs. calculated data (dots) by the GABP–ANN model at $\dot{\varepsilon }$ of (**a**) 0.005 s${}^{-1}$, (**b**) 0.01 s${}^{-1}$, and (**c**) 0.0167 s${}^{-1}$ from 0° RD; (**d**) 0.005 s${}^{-1}$, (**e**) 0.01 s${}^{-1}$, and (**f**) 0.0167 s${}^{-1}$ from 45° RD; and (**g**) 0.005 s${}^{-1}$, (**h**) 0.01 s${}^{-1}$, and (**i**) 0.0167 s${}^{-1}$ from 90° RD.

**Figure 7:**
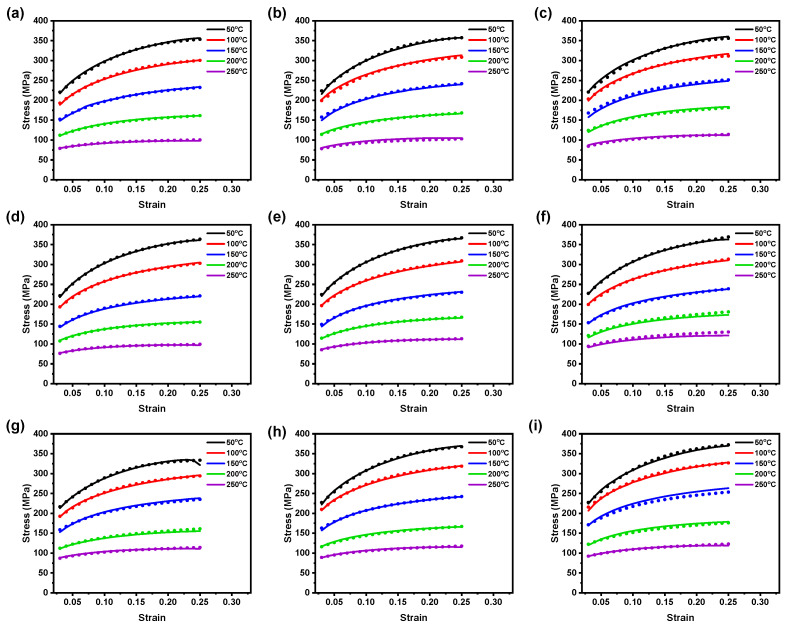
Comparison plot between test data (lines) vs. calculated data (dots) by the CFBP–ANN model at $\dot{\varepsilon }$ of (**a**) 0.005 s${}^{-1}$, (**b**) 0.01 s${}^{-1}$, and (**c**) 0.0167 s${}^{-1}$ from 0° RD; (**d**) 0.005 s${}^{-1}$, (**e**) 0.01 s${}^{-1}$, and (**f**) 0.0167 s${}^{-1}$ from 45° RD; and (**g**) 0.005 s${}^{-1}$, (**h**) 0.01 s${}^{-1}$, and (**i**) 0.0167 s${}^{-1}$ from 90° RD.

To assess the predictability of the proposed BP–ANN models, a comparison plot is established between the calculated data and the test data. This allows for a detailed examination of the model accuracy with respect to each individual experimental condition. The trained BP–ANN models performances such as simple BP–ANN, GABP–ANN, and CFBP–ANN models are further investigated by the graphical validations of comparing experimental observations against predicted data, as shown in Figure 5, Figure 6 and Figure 7. From Figure 5a–f, it can be noticed that the predicted data showed better agreement with the experimental observations, indicating that the simple BP–ANN model can be used to precisely forecast the flow stress at 0° and 45° RDs and to evaluate AZ31B magnesium alloy deformation behavior during the sheet-metal-forming process. However, the simple BP–ANN model, at 90° RD, could not represent the material deformation behavior at tested strain rates, as depicted in Figure 5g–i. Specifically, as depicted in Figure 5g–i, the trained BP–ANN model provided significant agreement with the test data at 0.01 s${}^{-1}$ (150 °C to 250 °C) and 0.0167 s${}^{-1}$ (150 °C) test conditions. Moreover, at 100 °C for the tested strain rates, the model showed considerable predictions against the experimental data; however, there were significant deviations in the predicted data observed in test conditions: 0.005 s${}^{-1}$ (50 °C to 250 °C), 0.01 s${}^{-1}$ (50 °C and 100 °C), and 0.0167 s${}^{-1}$ (50 °C, 200 °C and 250 °C). Similarly, Figure 6a–f also reveals a similar outcome to Figure 5a–f that the proposed GABP–ANN model was considerably similar to the test observations at 0° and 45° RDs. However, as illustrated in Figure 6g–i, the proposed GABP–ANN model also could not describe the material deformation at 90° RD for the test conditions of 0.005 s${}^{-1}$ to 0.0167 s${}^{-1}$ (50 °C to 250 °C). Moreover, as can be seen in Figure 6g–i, most of the calculated data points from the proposed GABP–ANN model fall away from the experimental observations and deliver a high prediction error compared with Figure 5g–i, which is obtained from the proposed BP–ANN model.

Figure 7 reveals the closeness of test data and predicted data from the optimized CFBP–ANN models for three rolling directions. From Figure 7a–i, it is noticeable that the predicted flow stress data from the optimized CFBP–ANN models falls close to the experimental observations at most of the test conditions for accounted rolling directions; however, for 90° RD, the optimized CFBP–ANN models demonstrated negligible deviations at the flow stress prediction at a strain rate of 0.0167 s${}^{-1}$ (250 °C for 45° RD and 150 °C for 90° RD), as depicted in Figure 7. In general, the optimized CFBP–ANN model outperformed both the basic BP–ANN and the optimized GABP–ANN models in effectively capturing the hot deformation flow behavior of the AZ31B magnesium alloy across a wide range of test conditions, as evidenced by the results presented in Figure 7. This indicates that the CFBP–ANN model is more capable of accurately describing and modeling the material’s behavior during hot deformation, making it a promising choice for studying such phenomena in various magnesium alloy materials.

**Figure 8:**
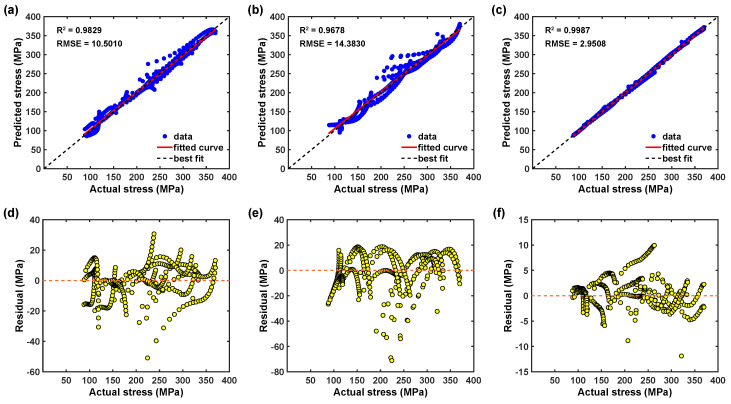
Correlation plots from 90° RD dataset: (**a**) BP–ANN model, (**b**) GABP–ANN model, and (**c**) CFBP–ANN model. Residual plots: (**d**) BP–ANN model, (**e**) GABP–ANN model, and (**f**) CFBP–ANN model.

**Figure 9:**
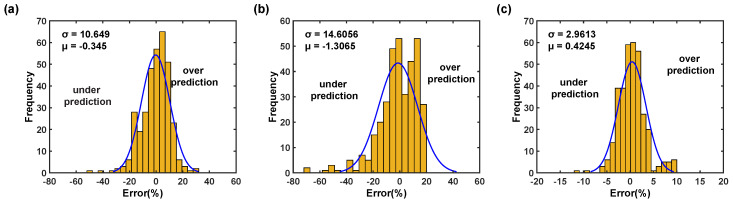
Statistical analysis of the relative error from 90° RD dataset: (**a**) BP–ANN model, (**b**) GABP–ANN model, and (**c**) CFBP–ANN model.

In addition, the proposed flow stress models are also graphically as well as numerically verified using the statistical metrics such as $R^{2}$ and RMSE, and the computed statistical parameters of the proposed hybrid artificial neural network models are summarized in Table 2. Figure 8a demonstrates that the predicted data from the trained BP–ANN model at 90° RD falls quite far away from the best fit line, and as listed in Table 2, the corresponding $R^{2}$ is determined to be 0.9829 with the prediction error value of 10.5010 MPa. This discussion indicates that the trained BP–ANN model does not describe the material deformation behavior effectively. Additionally, Figure 8d also supports the fact that the residual distribution is not random and confirms that the trained BP–ANN model has not achieved proper generalization and lacks model predictability. Moreover, Figure 9a illustrates that the trained BP–ANN model does both over and under the prediction of the flow stress, which results in prediction error ranges from −20 MPa to 20 MPa. Similarly, as demonstrated in Figure 8b, a noticeable number of the predicted data fall away from the best fit line for the optimized GABP–ANN model at 90° RD, and the corresponding $R^{2}$ for the model is estimated as 0.9678, which is considerably lower than the simple BP–ANN model. This coveys that the optimized GABP–ANN model has a better correlation than with the simple BP–ANN model. However, Figure 8e reveals that the residual distribution is still not random even though the model shows an improvement in the prediction, so it is also clear that the residual distribution is not random and confirms that the optimized GABP–ANN model has not achieved proper generalization and lacks model predictability.

**Figure 10:**
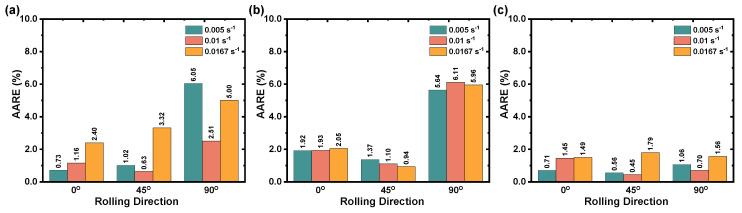
Prediction error from the proposed BP–ANN models: (**a**) BP–ANN model, (**b**) GABP–ANN model, and (**c**) CFBP–ANN model.

In addition, Figure 9b suggests that the optimized GABP–ANN model does both over and under the prediction of the flow stress the same as the simple BP–ANN model, which results in prediction error ranges from −40 MPa to 20 MPa. Figure 8c shows that the calculated flow stress using the optimized CFBP–ANN model at investigated deformation temperatures and strain rates mostly fall around the best fit line with a $R^{2}$ value of 0.9987 and confirm that the correlation value is higher than that of the simple BP–ANN and the GABP–ANN models. Moreover, Figure 8f also reveals that the residuals are randomly distributed and confirms the fact that the optimized CFBP–ANN achieved good generalization and stabilization during the network training process. Moreover, Figure 9c also confirms that the prediction error is reduced and falls within the range of −5 MPa to 5 MPa. The prediction errors between test observations and calculated flow stress were determined using Equation (5) and drawn as illustrated in Figure 10 [31]. Figure 10 clearly shows the performance of the proposed simple BP–ANN and optimized BP–ANN models. As reported in Figure 10a,b, both simple BP–ANN and optimized BP–ANN with a genetic algorithm (called GABP–ANN) models could significantly describe the deformation flow behavior at 0° and 45° RDs; however, these two models could not sufficiently describe AZ31B magnesium alloy flow behavior at 90° RD. On the other hand, Figure 10c demonstrates that the predicted values from the optimized CFBP–ANN model can forecast a more accurate prediction of flow stress across the entire test range and in three rolling directions.

The prediction errors of the proposed flow stress models have been compiled and presented in the Table 3. The results clearly indicate that the optimized CFBP–ANN model’s prediction errors range from 0.933% to 1.217%. In contrast, the basic BP–ANN model shows prediction errors in the range of 1.427% to 4.519%, and the optimized GABP–ANN model exhibits the lowest prediction errors, falling between 1.138% and 5.904%. These findings strongly suggest that the optimized CFBP–ANN model outperforms the other flow stress models in effectively forecasting the material’s deformation flow behavior. The CFBP–ANN model consistently achieves more accurate predictions over a wide range of test conditions, as evidenced by the lower prediction errors compared to the other models.

**Figure 11:**
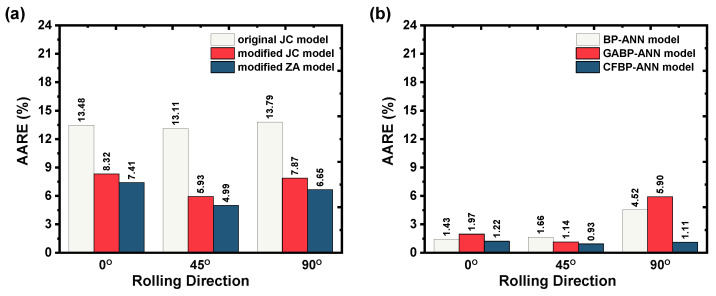
Graphical validation of proposed flow stress models: (**a**) developed conventional models and (**b**) proposed machine learning models.

Furthermore, Figure 11a represents the computed prediction error of the conventional models and indicates that the developed conventional models demonstrate a high prediction error throughout the test conditions considering three rolling directions. Moreover, Figure 11 strongly reveals that the proposed flow stress models based on basic and hybrid machine learning techniques better predict the flow stress. The Figure 11a to Figure 11b comparison confirms that the optimized CFBP–ANN models could track the AZ31B magnesium alloy flow behavior accurately across the accounted deformation temperatures and strain rates. Additionally, as summarized in Table 3, the prediction error from the proposed hybrid BP–ANN models also supports the fact that the optimized CFBP–ANN models could represent the AZ31B magnesium alloy flow behavior accurately. Overall, the above discussions related to the proposed hybrid BP–ANN models suggest that the recognized CFBP–ANN model demonstrated better predictability and can be practiced to explain and forecast the flow behavior of the AZ31B magnesium alloy.

## 5. Conclusions

The AZ31B magnesium alloy deformation behavior has been examined over a wide range of temperatures (50–250 °C) and strain rates (0.005–0.0167 s${}^{-1}$) by uniaxial warm tensile tests. It was identified that flow stress decreases with rising temperature and lowering strain rate in isothermal warm tensile deformation.

1. Based on the obtained flow stress data, a feed forward back–propagation artificial neural network (BP–ANN) model, a hybrid model with a genetic algorithm (GABP–ANN), and a constrained nonlinear function (CFBP–ANN) were investigated to forecast the AZ31B magnesium alloy deformation behavior.
2. The predicted data from the simple BP–ANN model demonstrated better agreement against the experimental observations and indicated that it can be devised to precisely predict the flow stress in the 0° and 45° rolling directions; however, in the 90° rolling direction, the proposed model cannot represent the material deformation behavior at tested strain rates. For example, the numerical quantifications such as $R^{2}$, 0.9829, and AARE, 4.5192%, from the 90° rolling direction, confirm that the simple BP–ANN model cannot represent the flow behavior accurately.
3. The GABP-ANN model also demonstrated that the optimized GABP-ANN model cannot represent AZ31B magnesium alloy deformation behavior at the tested strain rates. Moreover, incorporating a genetic algorithm with the BP-ANN model also failed to improvise the model performance and resulted in a high prediction error of 5.9037% for the 90° rolling direction, indicating that the GABP–ANN model lacks the model predictability of the BP–ANN model.
4. The optimized CFBP–ANN models can track the AZ31B magnesium alloy flow behavior accurately across the investigated deformation temperatures and strain rates. Additionally, the prediction error from the proposed hybrid BP–ANN models also confirms that it could capture AZ31B magnesium alloy flow behavior accurately as the prediction errors fall within the ±5 MPa range.

The discussions related to the proposed hybrid BP–ANN models indicate that the established CFBP–ANN model exhibited better predictability and can be practiced to explain and forecast the flow behavior of the AZ31B magnesium alloy accurately.

## Figures and Tables

**Table 1:** Chemical composition of AZ31B magnesium alloy (in wt%) [34].

Mg	Al	Zn	Mn	Si	Cu	Ca	Fe	Ni
Balance	2.50–3.50	0.60–1.40	0.20	0.10	0.05	0.04	0.005	0.005

**Table 2:** Estimated statistical metrics from the proposed BP–ANN model.

Angle	Conditions	BP–ANN Model		GABP–ANN Model		CFBP–ANN Model	
		$R^{2}$	Overall–$R^{2}$	$R^{2}$	Overall–$R^{2}$	$R^{2}$	Overall–$R^{2}$
**0°**	**0.005 s${}^{-1}$**	0.9996	0.9988	0.9978	0.9973	0.9996	0.9990
	**0.01 s${}^{-1}$**	0.9991		0.9972		0.9991	
	**0.0167 s${}^{-1}$**	0.9984		0.9970		0.9982	
**45°**	**0.005 s${}^{-1}$**	0.9995	0.9983	0.9993	0.9991	0.9998	0.9993
	**0.01 s${}^{-1}$**	0.9996		0.9991		0.9998	
	**0.0167 s${}^{-1}$**	0.9981		0.9991		0.9989	
**90°**	**0.005 s${}^{-1}$**	0.9836	0.9829	0.9710	0.9678	0.9990	0.9987
	**0.01 s${}^{-1}$**	0.9954		0.9696		0.9996	
	**0.0167 s${}^{-1}$**	0.9841		0.9664		0.9978	

**Table 3:** Estimated prediction error from the proposed BP–ANN model.

Angle	AARE (%)		
	BP–ANN Model	GABP–ANN Model	CFBP–ANN Model
**0°**	1.4269	1.9657	1.2172
**45°**	1.6587	1.1377	0.9327
**90°**	4.5192	5.9037	1.1081

## Data Availability

Not applicable.

## Abbreviations

The following abbreviations are used in this manuscript:

JC	Johnson–Cook
ZA	Zerilli–Armstrong
BP–ANN	Backpropagation artificial neural network
GA	Genetic algorithm
FE	Finite element
HN	Hidden layer neurons
IN	Input layer variables
NT	Number of training data
NO	Output layer variables
IW${}_{\mathrm{lb}}$	Lower bound limit of weights in hidden layer
IW${}_{\mathrm{ub}}$	Upper bound limit of weights in hidden layer
LW${}_{\mathrm{lb}}$	Lower bound limit of weights in output layer
LW${}_{\mathrm{ub}}$	Upper bound limit of weights in output layer
b1${}_{\mathrm{lb}}$	Lower bound limit of biases in hidden layer
b1${}_{\mathrm{ub}}$	Upper bound limit of biases in hidden layer
b2${}_{\mathrm{lb}}$	Lower bound limit of biases in output layer
b2${}_{\mathrm{ub}}$	Upper bound limit of biases in output layer
$R^{2}$	Coefficient of determination
AARE	Average absolute relative error
RMSE	Relative mean square error
